# Supplementary material for: Endogenous VIP VPAC1 Receptor Activation Modulates Hippocampal Theta Burst Induced LTP: Transduction Pathways and GABAergic Mechanisms
Source: Biology (Basel). 2022 Apr 20;11(5):627. doi: 10.3390/biology11050627 (PMC9138116; doi:10.3390/biology11050627)
Supplement: Supplementary file 1 [file biology-11-00627-s001.zip › Supplementaryfiles1-3-Caulino-Rocha2022BiologyWB.pdf]

***Supplementary file S1 - Western blots GluA1 –** order of samples is membranes from: control slices (C), VPAC1 antagonist (PG), theta-burst (T), and VPAC1 antagonist + theta-burst (PG+T), except where otherwise indicated. Due to incomplete records in some experiments, we can no longer identify the exposure times used for analysis. Experiments were developed on film thus MW markers are not visible. Different experiments are outlined in yellow.*

Exp 1 (different exposure times)

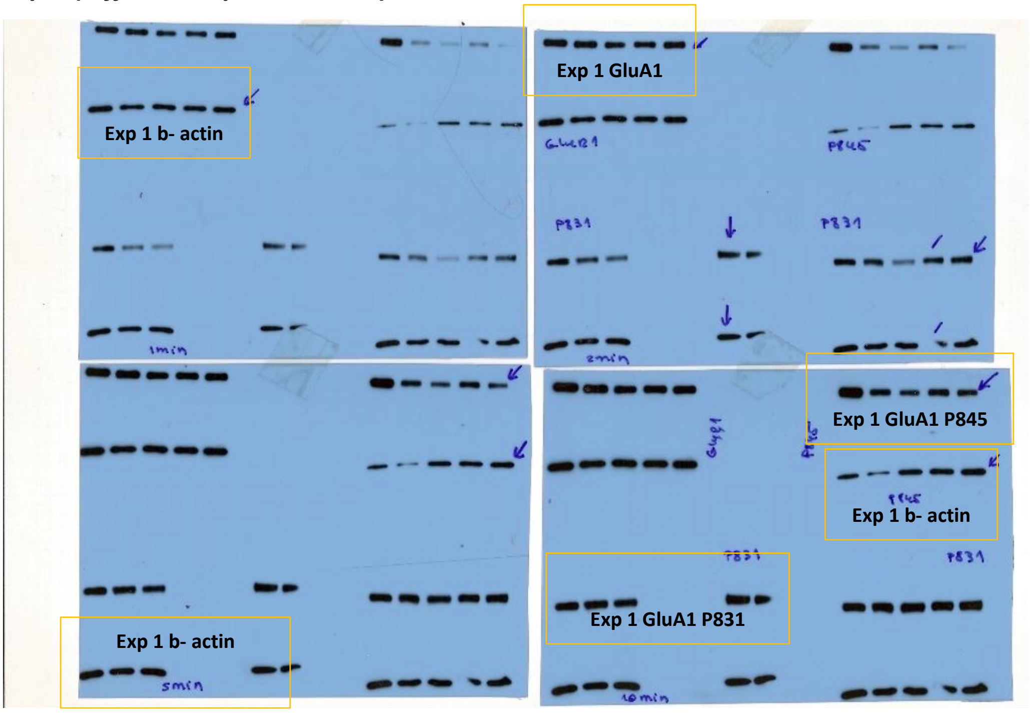

Exp 1 and 5 (different exposure times)

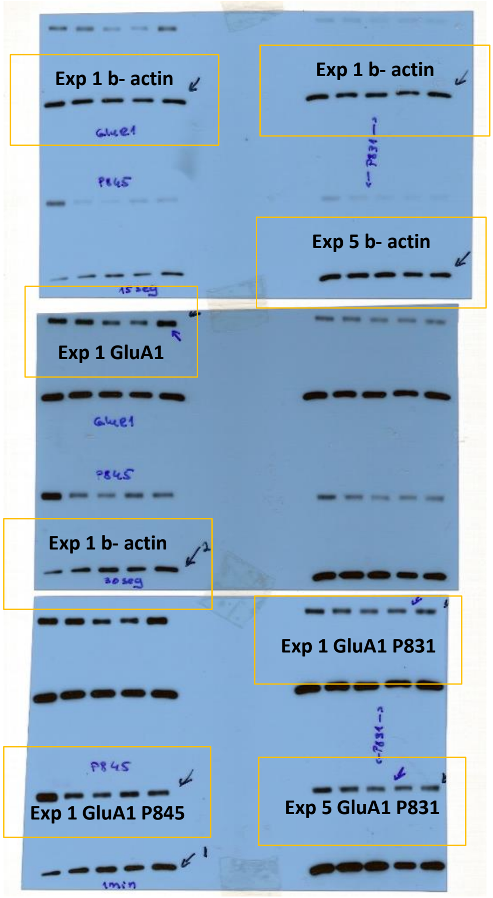

Exp 2 and 5 (different exposure times)

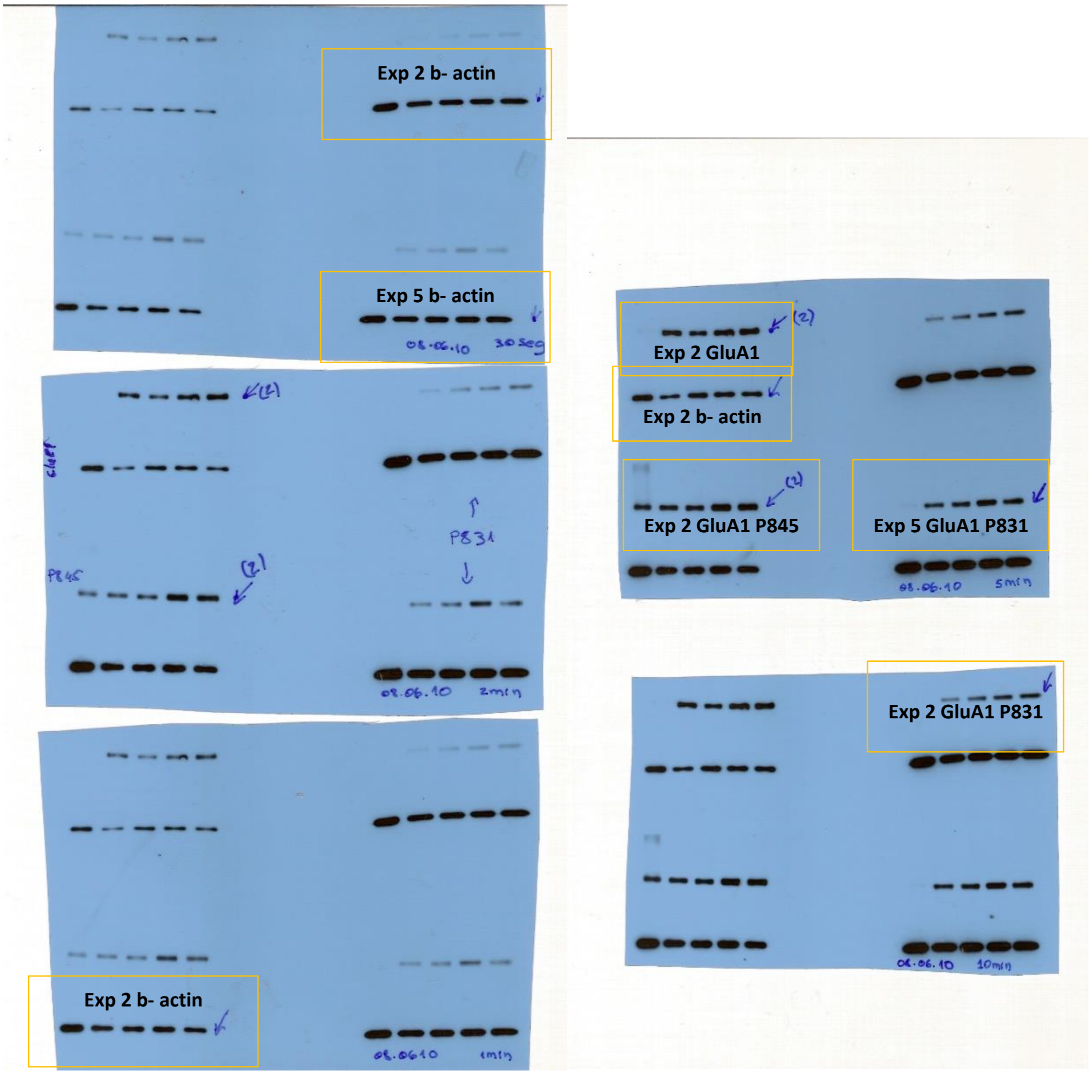

Exp 2 (different exposure times)

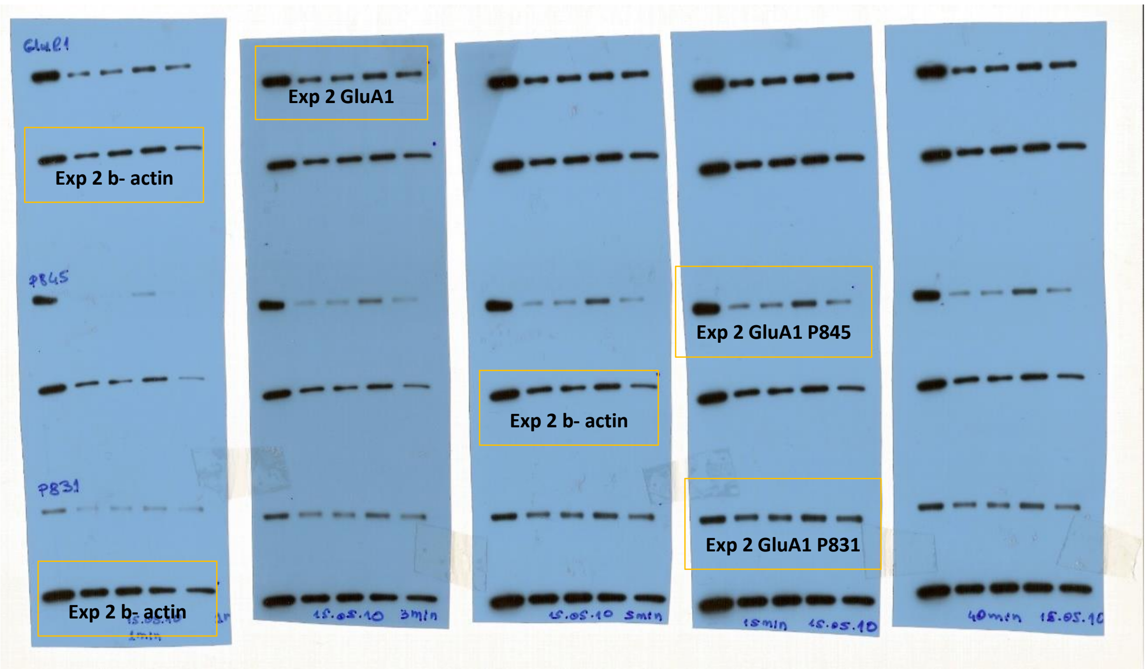

Exp 3 (different exposure times)

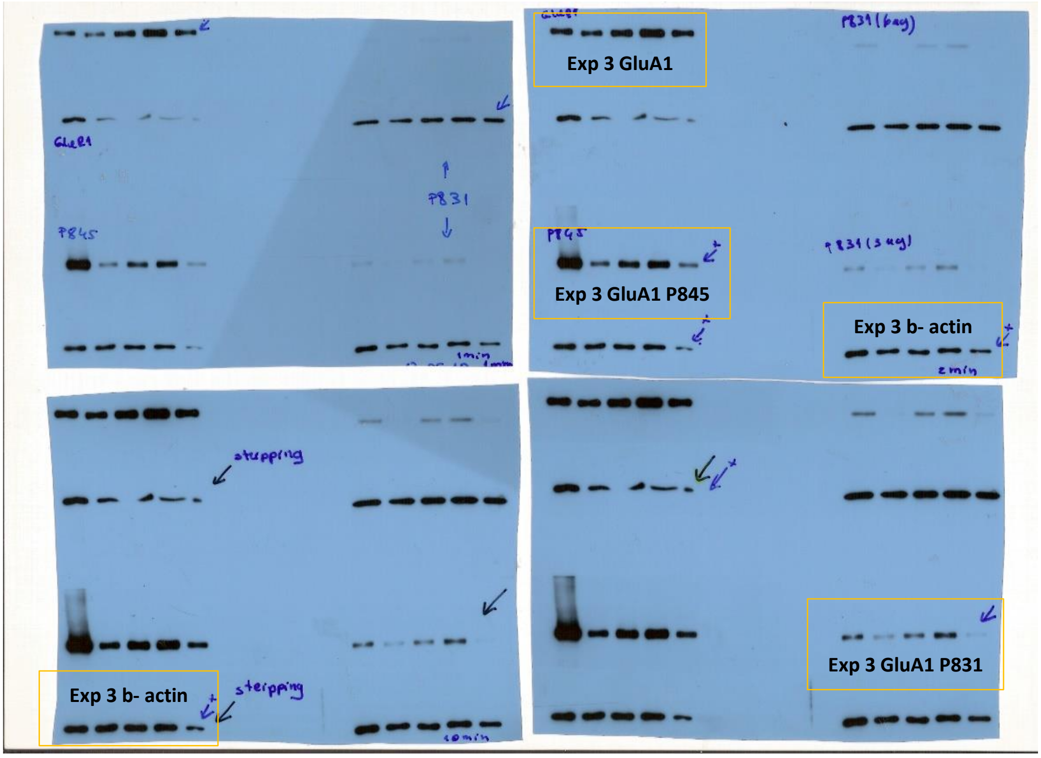

Exp 3 and 5 (different exposure times)

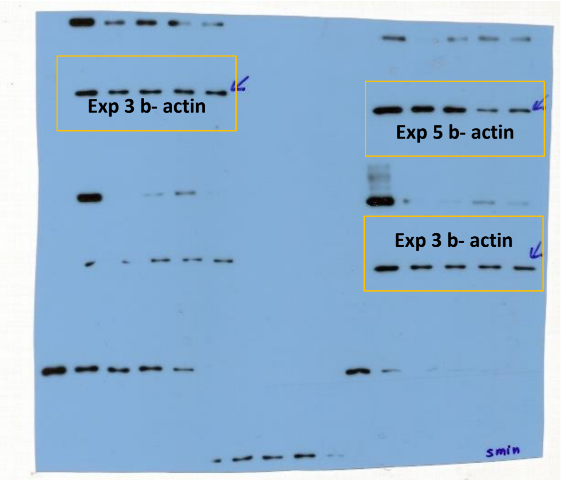

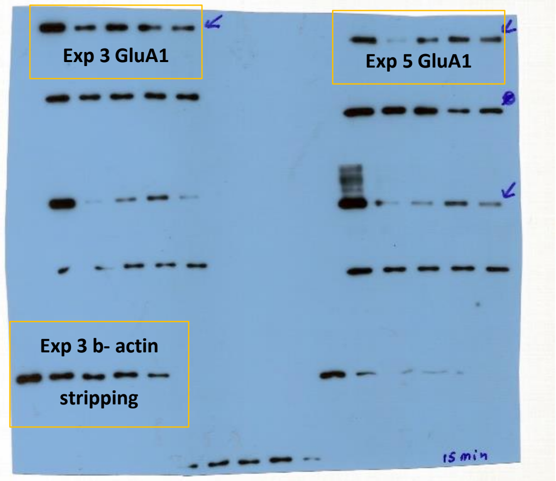

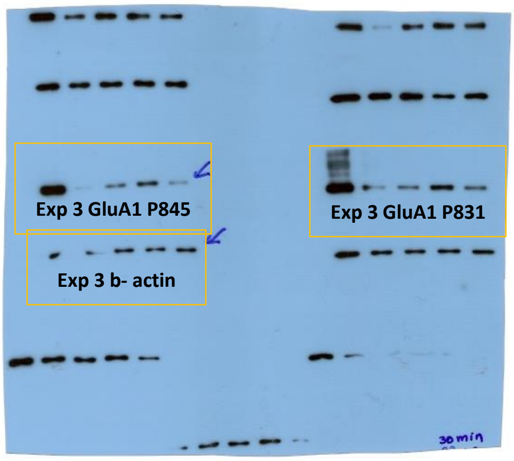

Exp 4 and 5 (different exposure times)

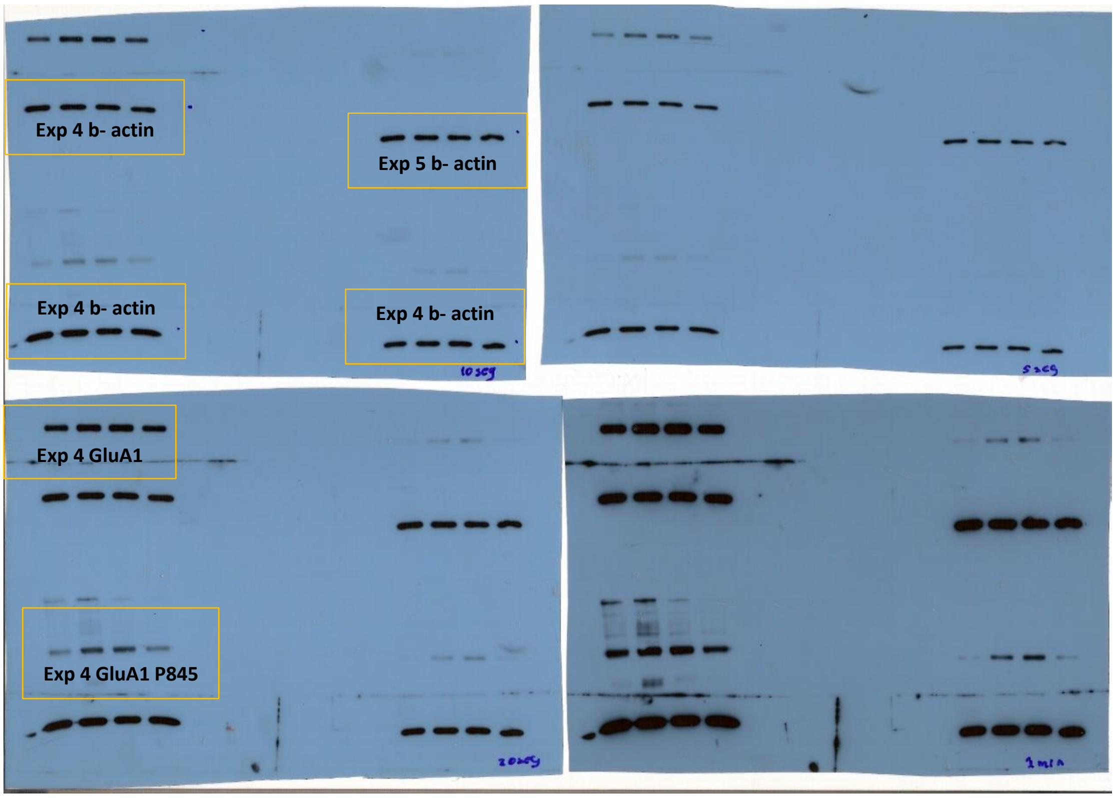

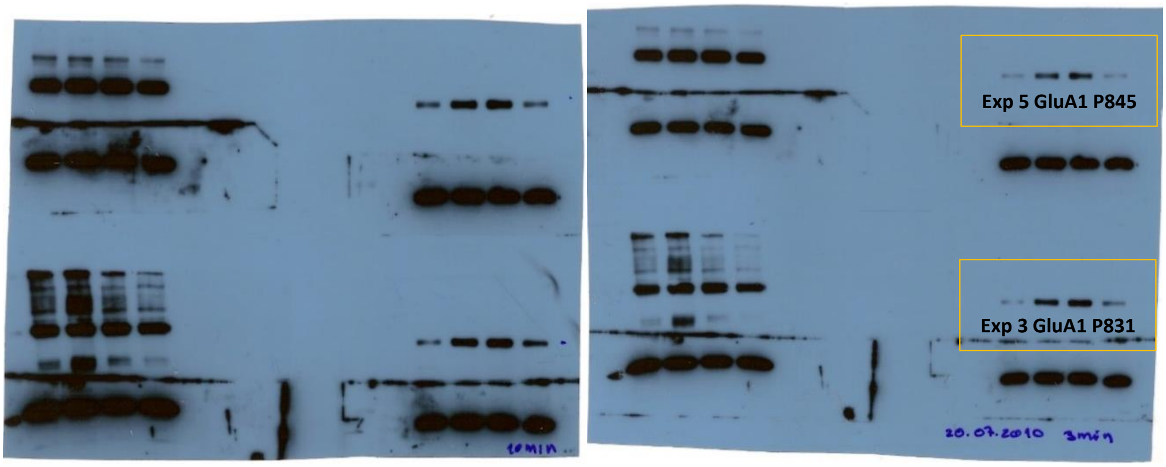

Exp 4 and 5 (different exposure times)

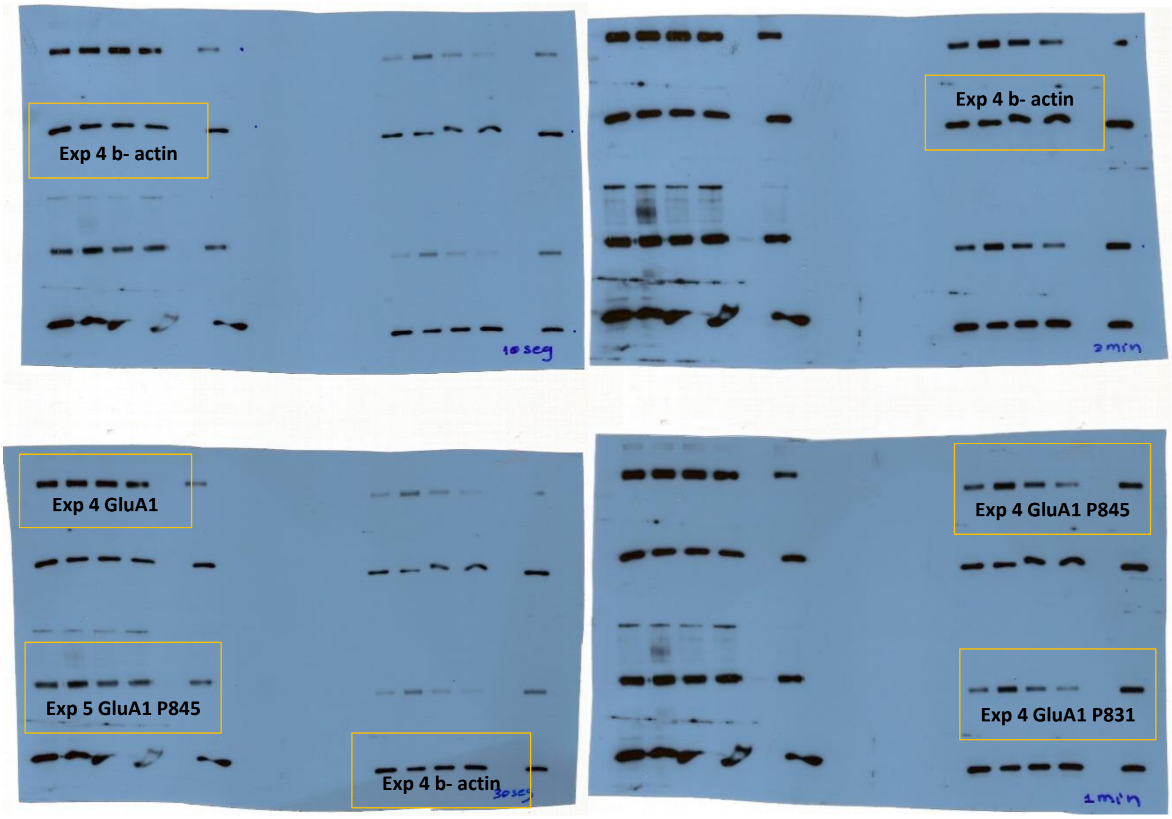

Supplementary file S2 - Western blots CaMKII *– order of samples is membranes from: control slices (C), theta-burst (T), VPAC1 antagonist (PG), and VPAC1 antagonist + theta-burst (PG+T), except where otherwise indicated. Different experiments are outlined in yellow.*

CamKII PThr286

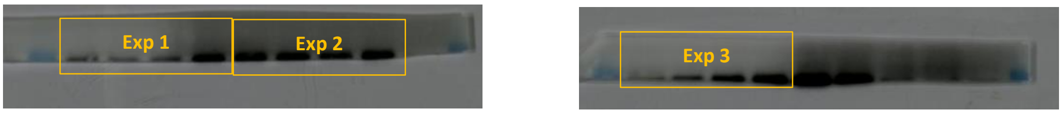

Obs: No staining on lower membrane when probed for CaMKII/CamKII PThr286

CaMKII (stripping)

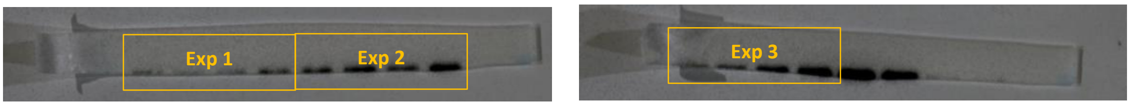

beta-actin M1

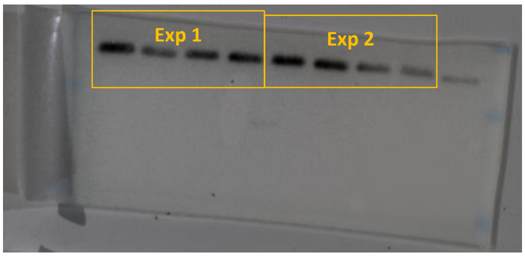

beta-actin M2

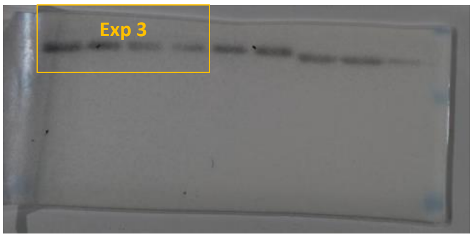

CamKII PThr286

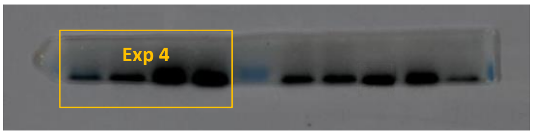

reversed order

CaMKII (stripping)

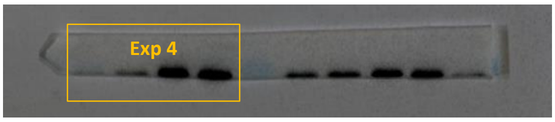

reversed order

beta-actin

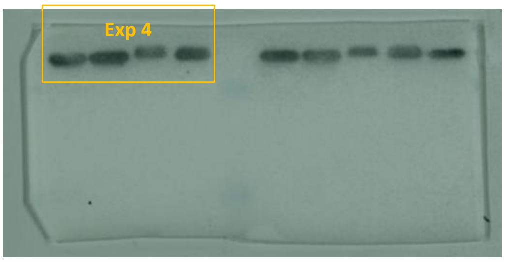

CamKII PThr286

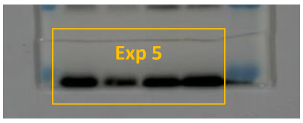

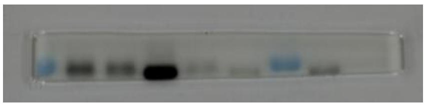

CaMKII (stripping)

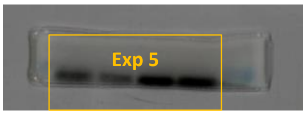

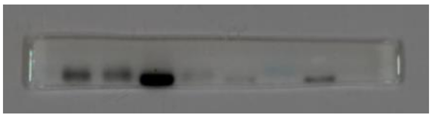

beta-actin

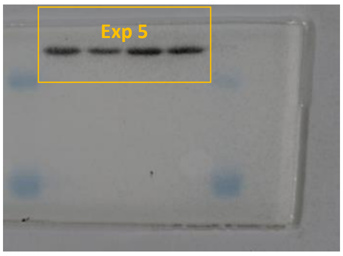

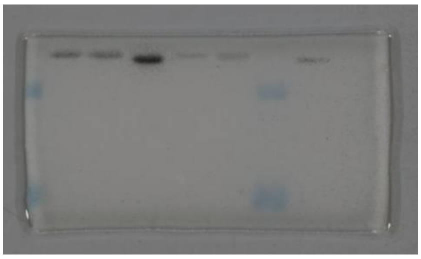

Supplementary file S3 - Western blots Kv4.*2 – order of samples is membranes from: control slices (C), theta-burst (T), VPAC1 antagonist (PG), and VPAC1 antagonist + theta-burst (PG+T), except where otherwise indicated. Different experiments are organized in a table.*

Experiment	***Kv***<br>***β-actin***	***PSer438***<br>***β-actin***	***P Thr602***<br>***β-actin***	***P Thr607***<br>***β-actin***
Exp1 – 08-11-2017<br>Order C and T	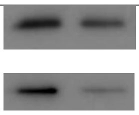 x x<br>x x	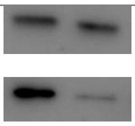 x x<br>x x	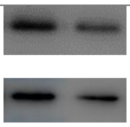 x x<br>x x	x
Exp1 –04-12-2017	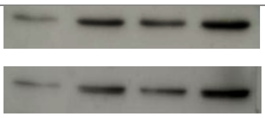	x x 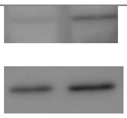<br>x x	x	x x 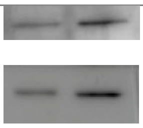<br>x x
Exp1 – 14-12-2017	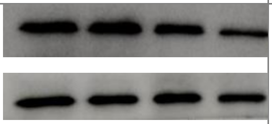	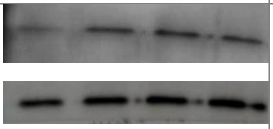	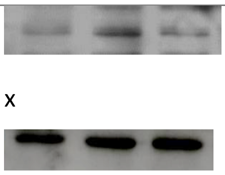 x<br>x	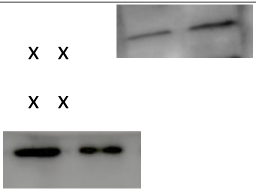 x x<br>x x
Exp 1 - 29-11-2018<br>Order C PG T and PG+T	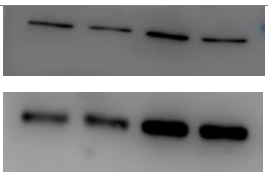	x	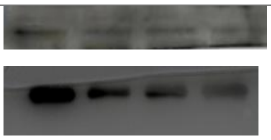	x
Exp2 – 30-11-2017 and 06-12-2017	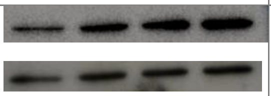	x	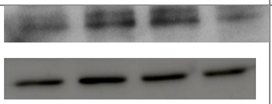	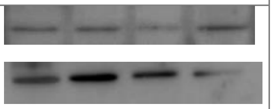
Exp2 – 14-11-2018<br>-> Order C PG T and PG+T	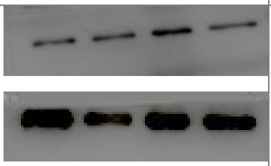	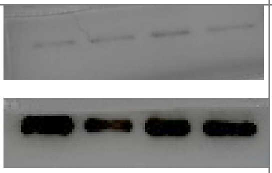	x	x
Exp 3 – 23-11-2017	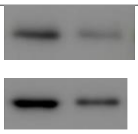	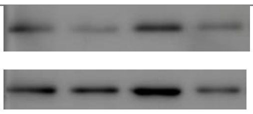	x x 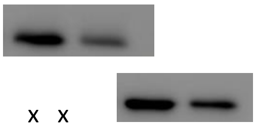<br>x x	x x 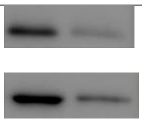<br>x x
Exp3 – 11-12-2017	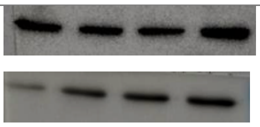	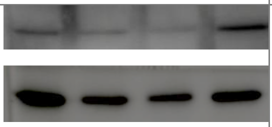	x	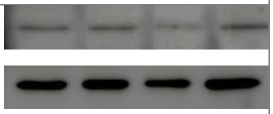
Exp4 – 06-12-2017	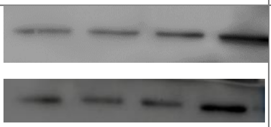	x	x	x
Exp5 – 13-12-2017	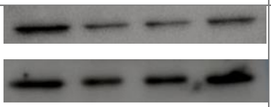	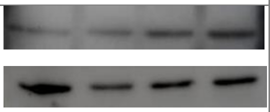	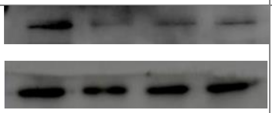	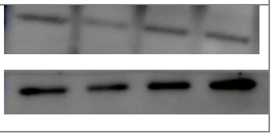

Optimization:<br><br>NOTE: images do not always have the MW marker in the immediate next lanes, i. e., samples from other projects were run in the same gel and were not included to avoid confusion.	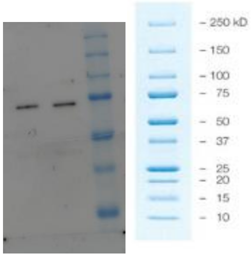	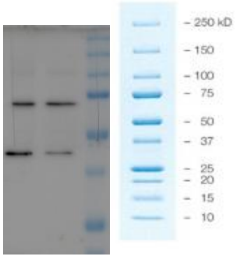<br>Lower half cut beta-actin	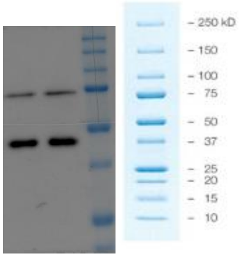<br>Lower half cut beta-actin	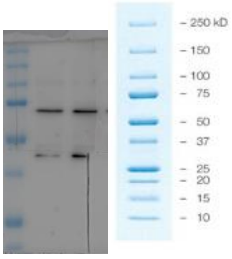<br>Lower half cut beta-actin
